# Characterization of Th2 Serum Immune Response in Acute Appendicitis

**DOI:** 10.3390/ijms27020733

**Published:** 2026-01-11

**Authors:** Nuno Carvalho, Jani-Sofia Almeida, Elisabete Carolino, Francisco Lopes, Susana Henriques, João Vaz, Hélder Coelho, Paulo Rodrigues dos Santos, Manuel Santos Rosa, Luís Moita, Carlos Luz, Paulo Matos da Costa

**Affiliations:** 1 Serviço Cirurgia Geral, Hospital Garcia de Orta, Unidade Local de Saúde Almada-Seixal, 2805-267 Almada, Portugal; franciscoa1424@gmail.com (F.L.); susanahenriques@campus.ul.pt (S.H.); jvaz1@campus.ul.pt (J.V.); carlos.luz@ulsas.min-saude.pt (C.L.); 2 Faculdade Medicina, Universidade Lisboa, 1649-028 Lisboa, Portugal; paulomatoscosta@gmail.com; 3 Laboratory of Immunology and Oncology, Center for Neuroscience and Cell Biology [CNC], University of Coimbra, 3004-504 Coimbra, Portugal; janisofiaalmeida@hotmail.com (J.-S.A.); paulo.santos@fmed.uc.pt (P.R.d.S.); 4 Institute of Immunology, Faculty of Medicine [FMUC], University of Coimbra, 3000-504 Coimbra, Portugal; msrosa@fmed.uc.pt; 5 Center of Investigation in Environment, Genetics and Oncobiology [CIMAGO], Faculty of Medicine, University of Coimbra, 3000-548 Coimbra, Portugal; 6 Coimbra Institute for Clinical and Biomedical Research [iCBR], Faculty of Medicine, University of Coimbra, 3000-548 Coimbra, Portugal; 7 Center for Innovation in Biomedicine and Biotechnology [CIBB], University of Coimbra, 3000-504 Coimbra, Portugal; 8 Clinical Academic Center of Coimbra [CACC], 3004-561 Coimbra, Portugal; 9 H&TRC—Health & Technology Research Center, ESTeSL—Escola Superior de Tecnologia da Saúde, Instituto Politécnico de Lisboa, 1990-096 Lisboa, Portugal; etcarolino@estesl.ipl.pt; 10 Serviço de Anatomia Patológica, Hospital Garcia de Orta, Unidade Local de Saúde Almada-Seixal, 2805-267 Almada, Portugal; hmoc85@gmail.com; 11 Innate Immunity and Inflammation Lab, Instituto Gulbenkian de Ciência Oeiras, 2780-156 Oeiras, Portugal; lmoita@igc.gulbenkian.pt; 12 Instituto de Histologia e Biologia do Desenvolvimento, Faculdade Medicina [FMUC], Universidade Lisboa, 1649-028 Lisboa, Portugal

**Keywords:** Acute Appendicitis, allergy, basophils, eosinophils, humoral immune response, hypersensitivity type I reaction, IgE, IL-4, IL-5, IL-13, Th2 cells

## Abstract

Acute Appendicitis (AA) is the commonest abdominal digestive surgical emergency, but its etiology is not clarified. Based on histologic observations, an allergic cause has been proposed. In a type I hypersensitivity allergic reaction, there is a Th2 immune response characterized by Th2 cells, eosinophils, basophils, IgE, IL-4, IL-5, and IL-13 serum elevation. Recent studies showed a local appendicular endoluminal and parietal Th2 immune response in acute phlegmonous appendicitis. We performed a prospective single-center study where we evaluated the Th2 blood immune response in 38 patients with acute phlegmonous appendicitis, 27 patients with acute gangrenous appendicitis, and 18 patients with the clinical picture of AA, who underwent appendectomy but had negative histology for AA (negative appendectomy group). Higher levels of basophils were found in phlegmonous appendicitis (*p* = 0.03), and higher levels of eosinophils were found in the control group (*p* = 0.003). Effector memory CD4 T cells re-expressing CD45RA were higher in gangrenous (*p* = 0.020) and central memory CD4 T cells in phlegmonous appendicitis (*p* = 0.004). The number of Th2 circulating cells was higher in gangrenous appendicitis (*p* = 0.037), while Th1 circulating cells were higher in phlegmonous appendicitis (*p* = 0.028). IL-4 blood concentrations were elevated in acute gangrenous appendicitis (*p* = 0.029). No significant differences were found in the levels of IgE, IL-5, or IL-13 in any of the groups. Thus, a Th2 response was not detected in patients’ serum with phlegmonous appendicitis. Serum levels of IgE, IL-5, and IL-13 were not different among patients with acute phlegmonous appendicitis, acute gangrenous appendicitis, and the negative appendectomy group. These findings are in contrast to our previous work in which we evaluated the Th2 response at the local level, at the appendicular luminal aspect and appendicular wall, in phlegmonous appendicitis and control groups, and we unequivocally showed a Th2 response in phlegmonous appendicitis. Thus, in patients with phlegmonous appendicitis, the local Th2 response is not reflected in the serum levels of immune cells and cytokines.

## 1. Introduction

Acute Appendicitis (AA) is the commonest abdominal surgical emergency worldwide [1,2]. Regrettably, this condition remains poorly understood [2,3]. It is proposed that luminal obstruction causes AA; however, this is seldom found [4,5].

Recently allergic factors have been advanced as etiologic factors of AA [6,7].

In fact, AA presents histological features of an allergic reaction characterized by eosinophil infiltration, perivascular histiocytic proliferation, and edema of germinative centers [8].

Thousands of antigens go through the digestive tract on a daily basis, and sometimes the immune system reacts against harmless antigens. This reaction can happen in any part of the digestive tract, but the appendix, due to its small diameter, has a limited capacity to accommodate edema, and ischemia of the mucosa will ensue, leading to AA [9,10].

Allergy is a type I hypersensitivity reaction involving Th2 cells that produce Interleukin (IL)-4, IL-5, IL-9, and IL-13. IgE is the immunoglobulin paradigm of allergy [11], with IL-4 and IL-13 as the main stimulators of IgE production [12]. IL-5 plays a pivotal role in eosinophil inflammation, as eosinophils are end-stage effector cells of allergy [13,14,15].

Basophils are fundamental for Th2 immune responses and the production of IL-4 and IL-13 [15,16,17].

Recent work shows the presence of a local allergy reaction in acute phlegmonous appendicitis (APA), with an elevation of Th2 cytokines IL-4, IL-5, and IL-9 in appendicular lavage fluid (ALF), a new concept for evaluating the local immune response in AA developed by our group [18].

An immunohistochemistry study of appendicular specimens, using a monoclonal antibody anti-IgE, showed higher levels of IgE in phlegmonous appendicitis than in the control group, corroborating the existence of a local allergy in APA [19].

It is a matter of controversy whether APA and acute gangrenous appendicitis (AGA) are distinct entities or a *continuum* of manifestations, with progression from phlegmonous to gangrenous appendicitis [18,19,20,21,22].

There is evidence of a local immune reaction in APA compatible with an allergic component [18,19]. These alterations are not seen in AGA [18,19].

Recent works point to two different entities: phlegmonous appendicitis with a Th2 response [18,19] and gangrenous appendicitis with Th1 and Th17 responses [20,21,22]. However, these results are controversial and not universally observed [23]. We have previously demonstrated the presence of a Th2 local immune response in APA [18,19].

Only a modest number of studies have been conducted to explore the role of plasma cytokines and lymphocyte populations in the field of AA.

Herein, we evaluate the systemic blood immune Th2 response in a group of patients with a clinical diagnosis of AA who were submitted to appendectomy.

If AA is a type I hypersensitivity allergic reaction, we would therefore expect that Th2 cells, eosinophils, basophils, IL-4, IL-5, IL-13, and IgE blood concentrations rise in APA, but not in AGA or the appendectomy negative group [24].

### Objective

In previous works, we showed a Th2 local immune response in the appendicular specimens of patients with Acute Appendicitis.

The present study aimed to evaluate the Th2 blood immune response by measuring Th2 cells, eosinophils, basophils, IL-4, IL-5, IL-13, and IgE serum concentrations in patients with a clinical diagnosis of Acute Appendicitis.

## 2. Results

### 2.1. Patients’ Characteristics

The study group included 83 patients, 53 males and 30 females. There were 65 patients with a histological diagnosis of AA; of those, 38 with APA and 27 with AGA, and 18 patients with a clinical diagnosis of AA, but with negative histology (NPA).

Regarding age, gender, BMI, and presence of allergies, no statistically significant differences were detected between the three histological types (Table 1).

A significant difference was found regarding the time to find medical consultation with the shortest time in APA.

Allergy was present in thirteen patients (15.6%): allergic rhinitis in three cases, penicillin allergy in five cases, and one case each of ceftazidime, cefuroxime, metronidazole, methimazole, and nickel allergies.

### 2.2. Hematologic Parameters

For WBC, no difference was found between the histological groups (Table 2).

The highest levels of neutrophils and CRP were present in AGA. The lowest levels of lymphocytes and eosinophils were present in AGA. Concerning basophils, the highest levels were present in APA (Table 2).

### 2.3. Flow Cytometry Population Levels

#### 2.3.1. T Cell, B Cell, and NK Cell Flow Cytometry

For lymphocyte populations, T cells, B cells, and NK cells, no differences were found (Supplementary Table S1).

#### 2.3.2. TCD4 and TCD 8 Cell Flow Cytometry

For CD4 T and CD8 T cells, no differences were found, as well as for the activation marker HLA-DR (Supplementary Table S2). When evaluating the maturation stages of CD4 T cells, we looked for the expression of CD45RA and CCR7 molecules and four populations were identified: naïve (CD45RA+ CCR7+), central memory (CM) (CM, CD45RA−, CCR7+), effector memory (EM) (EM, CD45RA− CCR7−), and effector memory re-expressing (EMRA) CD45RA (EMRA, CD45RA+, CCR7−). No differences were found for naïve and EM CD4 T cells (Table 3).

For CM CD4 T and EMRA CD4 T cells, the highest levels were present in APA (Table 3).

#### 2.3.3. Th1, Th2, Th17, Treg Flow Cytometry

For Treg cells, no differences were found (Supplementary Table S3). Differences were found in the Th1 and Th2 cell populations, with the highest levels being present, respectively, in APA and AGA (Table 4).

For the population of Th17, no differences were found (Table 4). For HLA-DR+ Th cells, no differences were found (Supplementary Table S3).

### 2.4. Serum IL-4, IL-5, and IL-13 Levels

Cytokine’s serum, IL-4, IL-5, and IL-13 levels and IgE levels are presented in Table 5.

IL-5 was present in the blood of all the studied subjects. For IL-4 and IL-13, blood levels were undetectable, respectively, in eleven and six patients: IL-4 was undetectable in two NPA patients, six APA patients, and three AGA patients, and IL-13 was undetectable in three APA patients and three AGA patients. The highest levels of IL-4 were present in AGA. 

IgE was elevated in 15,5% of the cases with a clinical diagnosis of AA.

No significant differences were found for IL-5, IL-13, and IgE blood levels and appendicular histology (Table 5).

### 2.5. Relationship Between Serum IgE, IL-4, and IL-13 Levels

No correlation was found between the blood levels of IgE and Th2 cytokines IL-4 and IL-13 (Table 6).

### 2.6. Relationship Between the Th2 Population, IL-4, IL-5, IL-13, and Serum IgE Levels

No correlation was found between the Th2 population, IL-4, IL-5, IL-13, and IgE serum levels (Table 6).

A greater IL-4 response is related to a greater IL-5 response (Table 6). No correlation was found between Th2, IL-4, IL-5, and IL-13 and eosinophils and basophils.

## 3. Discussion

The type I hypersensitivity allergic reaction [10] is driven by a Th2 response mediated by allergen-specific IgE [11] and involves eosinophils, basophils, IL-4, IL-5, IL-9, and IL-13 in the orchestration of this complex process [13,15,18,25,26,27].

Th2 immune responses are present in eosinophilic esophagitis [28]. The appendix is another digestive organ which can potentially be involved in a Th2-related disease.

Herein, we evaluate several aspects of the systemic immune response in AA.

### 3.1. Eosinophils

Eosinophilia is often present in allergic diseases [25,28]. In the current series of patients, the highest levels of eosinophils were present in NPA, and the lowest levels in AGA. A confounding factor for this result may be the huge degree of local inflammation that recruits/destroys these blood elements [29]. Sepsis is associated with eosinopenia, and the lowest levels of eosinophils in AGA may be attributed to a similar mechanism [29]. We showed that eosinophils infiltrate the appendicular wall in AA, with the greatest number in APA [30]. Recently, we also found eosinophil granule protein elevation in APA, both at local and systemic levels [31].

### 3.2. Basophils

Basophils are the least frequent granulocyte population, but they significantly contribute to Th2 cytokine-mediated inflammation [32]. Basophil levels in blood and tissues rise in Th2-mediated allergic diseases [26]. Mean basophil count is higher in APA, as was present in our study, corroborating the likelihood of a Th2 reaction in AA [33].

### 3.3. Cytokines

IL-4 and IL-13 are pleiotropic cytokines that act as growth factors for Th2 cells and are involved in the isotype switch from IgM to IgE [24,34,35,36]. IL-4 is the main cytokine that stimulates IgE production [11,24]. In our study, IL-4 was elevated in AGA, while IL-13 had a normal value. For IL-5, a cytokine that has a fundamental role in allergic inflammation, no differences were found between APA and AGA [13].

### 3.4. IgE

The final effector of the Th2 response is IgE. No significant differences were found between groups. There are several possible explanations for the absence of IgE elevation in allergy, such as the well-known fact that the majority of IgE antibodies are present on the mast cell surface and not in the blood [37]. Using a monoclonal antibody, we found elevated levels of IgE in appendicular specimens of APA compared with those of incidental appendectomy [19]. In asthma, the association with IgE levels is not uniform [38], and experimental asthma can be provoked in animals in the absence of IgE, which suggests that other pathways can induce allergy [39].

IgE elevation can occur in specific subtypes without repercussion in the total concentration [40]. We only evaluated total IgE; therefore, the absence of elevated IgE serum levels in AA does not exclude an allergic reaction. Further studies should focus on IgE and IgG subclasses.

IgE serum levels were evaluated in AA, recurrent appendicitis, and controls, including histologically normal appendices, healthy volunteers, and patients with asthma [41]. The higher levels of IgE were present in recurrent appendicitis [41]. The authors claimed that recurrent AA may be due to a predisposition to the type I hypersensitivity reaction [41]. Several previous studies showed elevated IgE levels in AA [42,43,44,45]. The risk of complicated appendicitis is three times lower in children with IgE-mediated allergy than in those without allergy, suggesting a protective effect of allergy for complicated AA [22]. This is in accordance with different etiologies for phlegmonous and gangrenous appendicitis, with different immunologic responses [18,19,20,21,22]. IgG is also involved in allergy, namely IgG4, which is not evaluated in the present study [46].

A study shows that IgA, IgE, IgG, and IgM serum levels, albeit without a significant difference, were higher in the control group, opposite to APA and AGA [47].

### 3.5. Lymphocytes

Lymphopenia is common in gangrenous appendicitis [48,49], as in our study, and is accompanied by an increase in lymphocyte numbers at the appendicular wall, demonstrating lymphocyte recruitment to the inflamed appendix [48].

We evaluated lymphocyte subsets, and, like others, we found no difference for the B cell population or for the CD4 and CD8 T cell subsets [48]. CD4 T cells are involved in allergy and adaptive immunity. CM T cells are antigen-specific memory cells that reactivate and expand by antigen exposure [50]. CM CD4 T cells were significantly elevated in APA. This can be interpreted as a response to previous antigen exposure.

The EMRA CD45RA T cell is associated with the expansion of a cytotoxic phenotype [51]. EMRA CD4 T cell levels were higher in AGA, which is compatible with their cytotoxic effect, associated with tissue destruction [51]. A previous study also showed EMRA CD45RA T cell elevation in the peripheral blood of patients with AA compared to the control [50], but no distinction was made for AGA or APA [48]

For naïve and EM CD4 T cells, no differences were observed, in contrast with another study that showed T memory cell depletion in peripheral blood and an increase in the inflamed appendix [48].

Naïve CD4 T cells differentiate into Th1, Th2, Th17, and Treg, depending on the cytokine environment [52].

### 3.6. Immune Response—A Look at a Glance

Recent evidence suggests the presence of a local Th2 immune response in APA [18,19]. It is expected that Th2 circulating blood levels will increase in the occurrence of an allergy [53]. In contrast, in our study, we found that Th2 levels were higher in AGA.

For Th1, the highest levels were present in APA. These results disagree with previous works that showed a Th1 response in AGA [20,22]. We can speculate on a possible role for circulating cytotoxic Th2 cells in AGA. A study that evaluated gene expression showed no Th1 or Th2 responses in AA [51].

For Th17 cells, no histological differences were found. Previous works showed that a Th17 profile was present in gangrenous appendicitis [21].

Th2 cells produce IL-4, IL-5, IL-13, and, through IL-4 and IL-13, induced IgE production [15]. No correlation was found between the blood levels of Th2 cells and Th2 cytokines IL-4, IL-5, IL-13, and IgE.

In the present study, patients came to the emergency department with a median of 24 h after the initial clinical manifestations. The half-life of Th2 cytokines is very short, so we can speculate that at the time that the blood serum was collected, their values may have fallen, as cytokines are transient and non-permanent products [35]. In fact, early or transient systemic immune responses may not be captured by the present study design.

A positive correlation was found for IL-4 and IL-5. This can be explained by the fact that IL-4 induces FcƐRI expression by human mast cells [hMCs], which augments hMC capacity to generate IL-5 [54].

Data provided by our own group [18,19,30,31,55,56] and others [20,21,22,23] showed a Th2 immune response at the local level in AA. 

Control group selection is a complex process. In the present study, patients diagnosed with suspected AA, but without histological confirmation, were the control group, which can induce a bias, as these patients can also have a systemic inflammatory response, not related to AA, and potentially attenuate detectable differences between groups. Other alternatives for a control group are not available.

We evaluated the Th2 immune response in serum in patients with APA and AGA and the negative appendectomy group, and globally, no Th2 response was present at the time of our blood collection.

It is well known that Th2 cell responses are mainly activated at affected sites [57]. In AA, the involved organ is small, and therefore, local alterations in the Th2 response may not have systemic repercussions. In a previous study, we demonstrated higher levels of Th2 cytokines, IL-4 and IL-5, in the appendicular lavage fluid in patients with APA than in those with AGA and NPA, which reflected the type of inflammatory local changes [18].

In allergic rhinitis, IgE elevation is present at the nasal mucosa but not in serum [58], a finding that agrees with the lack of elevated IgE in the serum of AA patients in our study. Eosinophilia, a common feature of allergy, depends on the size of the affected organ and is more common in asthma than in allergic rhinitis [59].

Strengths: The prospective design of this study, with clear histologic confirmation of AGA, APA, and NPA. This is the first report of a Th2 immune response evaluation in adult AA with simultaneous cellular, cytokine, and immunoglobulin determinations, specifically Th2 cells, eosinophils, basophils, IL-4, IL-5, IL-13, and IgE. We also provide pioneering data on lymphocyte populations in AA, which deserves to be expanded.

Limitations: This is a single-center study with a small group of patients and the absence of a control group, other than NPA. Total IgE and not serum-specific IgE were determined. The absence of sequential time measurements for evaluating serum dynamic changes is an important limitation. The results should be evaluated by others, as the medical literature is scarce on this topic. Future studies should provide a larger cohort of patients, ideally on a multi-center basis.

Our findings generate more questions than answers, and further studies are warranted.

Several points are proposed for future research:

Prospective multicenter studies with large sample sizes to improve statistical power and inclusion of pediatric patients, as this particular population has a high prevalence of allergic diseases [22,23].

Simultaneous evaluation of the Th2 response in the appendicular wall, appendicular lavage fluid, and serum to evaluate the distribution of the immune response in Acute Appendicitis in local and systemic compartments [18,19,60].

Evaluation of cytokine kinetics along a time course to clarify if transient serum elevations are present at a particular moment in AA natural history.

Serum specific IgE and IgG4 should be determined, due to its high specificity for the diagnosis of allergy [39,40,57].

Development of an AA laboratory model to test the immune response to several antigens.

If it is confirmed that AA is an allergic disease, a new era of therapeutic modalities will ensue [18].

## 4. Material and Methods

### 4.1. Study Population

As no available information was present in the current literature for sample size calculations, we performed a pilot study [61]. This single-center, prospective design study recruited patients aged above 18 years, from April 2016 to June 2017, who were evaluated in the emergency department of Hospital Garcia de Orta, with the clinical diagnosis of AA, and submitted to appendectomy. Exclusion criteria: patients aged up to 18 years, as they are taken care of by pediatric surgeons, and pregnant women, as pregnancy can induce modifications in the humoral immune response [62].

The study group was composed of patients with histologic features of AA and a control group of patients submitted to appendectomy for the presumption of AA, with appendicular specimens showing normal histological features.

All patients underwent surgery with general anesthesia and perioperative antibiotic treatment.

### 4.2. Setting

The study was carried out in a tertiary public hospital, with 600 beds, serving an urban population of 280,000 inhabitants.

### 4.3. Pathologic Analysis

Neutrophilic infiltration in the *muscularis propria* of the appendicular wall defines APA, and necrosis of the wall of the appendix in a background of transmural inflammation defines AGA [63]. If no neutrophil infiltrate was present in the *muscularis propria*, the specimen was classified as a non-pathologic appendix (NPA) [63,64]. The latter group, NPA, is frequently referred to as negative appendectomy [63]. A dedicated digestive pathologist blinded to the Th2 humoral immune response, and initial pathologic examination, evaluated all the appendicular specimens.

### 4.4. Th1, Th2, and Th17 Populations

Multiparametric flow cytometry analysis was carried out to measure the relative frequency of circulating Th1, Th2, and Th17 cells. Peripheral blood was collected in a 3 mL tube with 600 µL of Transfix stabilization reagent (Cytomark, Buckingham, London, UK). A hematological counter (A^c^T diff, Beckman Coulter, Pasadena, CA, USA was used for white blood cell (WBC) counting and 100 uL of fresh blood or up to 1 × 10^6^ WBC was incubated with extracellular monoclonal antibodies (mAbs) for 15 min in the dark at room temperature. Following incubation, erythrocytes were lysed with BD Lysing Solution (BD Biosciences, San Jose, CA, USA) for 10 min. Cell suspensions were centrifuged afterwards at 450× *g* for 5 min, the supernatant was discarded, and the pellet was washed by centrifugation with 2 mL of 1× PBS (phosphate-buffered saline) at 450× *g* for 5 min. Supernatant was thrown out and cells were re-suspended in 200 μL of 1× PBS and obtained in an eight-color flow cytometer BD FACSCanto II with BD FACSDiva software version 6.1.3 (BD Biosciences, San Jose, CA, USA).

**Figure 1:**
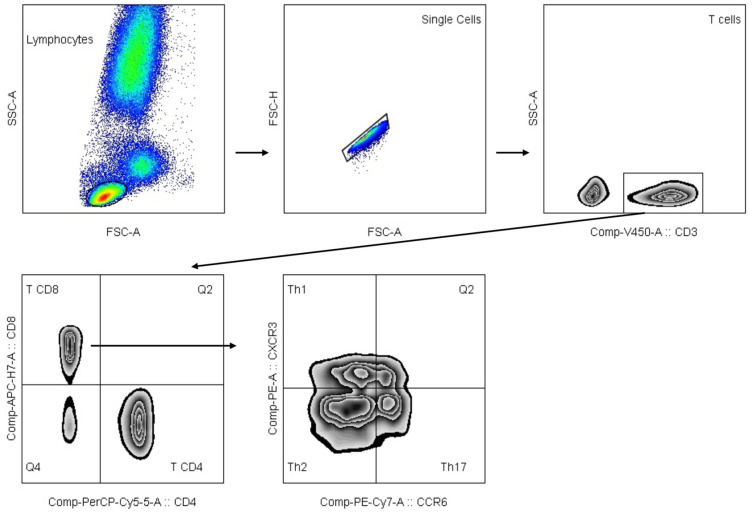
Image of the gating strategy applied for the identification of Th1, Th2, and Th17 populations obtained using multiparametric flow cytometry analysis of peripheral blood samples. Flow cytometry raw data (FCS files) were exported and analyzed using Flow-Jo v.10.7 software (BD Biosciences, Ashland, OR, USA). Lymphocytes were discriminated on morphologic parameters (SSC-A versus FSC-A), and doublets were excluded from the analysis (FSC-H versus FSC-A). Next, gated on single-cell lymphocytes, T cells were selected through a positive expression on the CD3 molecule. Subsequently, CD4 T and CD8 T cells, as well as double-positive (DP, CD4+, CD8+) and double-negative (DN, CD4−CD8−) T cells, were classified based on the expression of CD4 and CD8 molecules. To evaluate Th cells, the expression of CX3CR1 and CCR6 molecules was used to discriminate Th1 (CX3CR1+ CCR6−), Th2 (CX3CR1− CCR6−), and Th17 (CX3CR1− CCR6+) cells (CX3CR1− CCR6−).

Flow cytometry raw data (FCS files) were then exported and analyzed using FlowJo v.10.7 software (BD Biosciences, Ashland, OR, USA). A panel of mAbs (Supplementary File S1) was designed for the discrimination of B, T, and NK cells and the analysis of key receptors associated with maturation, differentiation, and activation [65]. Different mixes of monoclonal antibodies (mAbs) were used to identify T cells, B cells, NK cells, and subpopulations of CD4 T cells: (1) CD4 T cell maturation subsets; (2) helper T cells [Th1, Th2, and Th17; and (3) regulatory T (Treg) cells]. To assess the activation state of T cell subpopulations, the expression of the molecule HLA-DR on CD4 T, CD8 T, Th, and Treg cells was also evaluated. The gating strategy for T, B, and NK cells, CD4 T cell maturation subsets, and Treg cells is detailed in Supplementary File S2. The gating strategy for Th1, Th2, and Th17 cells is depicted in Figure 1. T, B, and NK cells are presented as % of lymphocytes. Th and Treg cells are presented as % of T CD4+ cells.

### 4.5. White Blood Cell Count

DxH 900 equipment (Beckman Coulter, Inc.Pasadena, CA, USA) was used for WBC count employing the Coulter principle. VCSn technology was used for evaluating differential WBC populations, and eosinophils and basophils were automatically shown in mm^3^. For accuracy and reproducibility, the count was made in triplicate.

Reference values for WBC are 4.00–11.00 × 10^9^/L, for neutrophils, 1.90–8.00 × 10^9^/L (40.0–74.0%), for lymphocytes, 0.90–5.20 × 10^9^/L (19.0–48.0%), for eosinophils, 0.0–7.0 × 10^9^/L (0.0–0.80%), and for basophils, 0.00–0.20 × 10^9^/L (0.0–1.5%).

### 4.6. IL-4, IL-5, and IL-13 Determinations

For IL-4, IL-5, and IL-13 determinations, blood samples were collected by venipuncture in vacutainers before anesthesia induction. Samples were centrifuged and 1 mL of the supernatant was collected and stored at −20 °C. IL-4, IL-5, and IL-13 levels were assessed by ELISA (human IL-4, human IL-5, and human IL-13 MAX, BioLegend, San Diego, CA 92121, USA) according to the manufacturer’s protocol. The cytokine levels were expressed in pg/mL. The lower limit of sensitivity is 4 pg/mL for both IL4 and IL-5 and 8 pg/mL for IL-13.

### 4.7. IgE Determinations

A 5 mL blood draw was harvested by venipuncture into vacutainers prior to anesthetic induction. Samples were centrifuged at 2500 rpm for 5 min, and serum was stored at −20 °C until processed as directed by the manufacturer. Quantitative determination of total IgE is based on a two-step solid-phase chemiluminescence immunoassay performed on a Siemens^®^ Immulite^®^2000XPi analyzer. The manufacturer’s reference range for IgE for healthy adults is <165 IU/mL.

### 4.8. Statistical Analysis

SPSS statistical software, V26.0 for Windows, and R statistical (RSStudio 4.3.3) software were used for analyzing the results. *p* < 0.05 was considered significant. The Shapiro–Wilk test was selected for testing the normality of the data. For sample presentation, frequency analysis was used for qualitative data, and for quantitative data, minimum, maximum, Mean, and standard deviation were used when the normality assumption was verified, otherwise, Median and interquartile range [Q1–Q3] were used. For the comparison of k > 2 independent groups, the One-Way ANOVA or the Kruskal–Wallis test were used when the assumption of normality was verified or when it was not verified, respectively. Tukey HSD multiple comparison tests were used for One-Way ANOVA, or the Kruskal–Wallis multiple comparison tests were used when statistically significant differences were found. To study the relationship between two quantitative variables, Spearman’s correlation coefficient was applied, since the assumption of normality was not verified. To study the relationship between two qualitative variables, the Chi-Square test by Monte Carlo simulation was used, since the applicability assumptions of the Chi-Square test were not verified. Multinomial Logistic Regression was applied; however, the results are not presented since they were not significant as expected considering the small number of patients in each histological category.

### 4.9. Other Data

Other data were evaluated, like patient age, sex, presence of co-morbidities, clinical history onset and their duration, presence or absence of peritonitis, open or laparoscopic appendectomy, intra-operative details, complications, hospital length of stay, and other histologic features. Individuals were also asked about allergic disorders.

### 4.10. Ethical Considerations

All the patients gave their written informed consent to participate in the study. The confidentiality of the information is always maintained, and anonymity is preserved. No potentially identifiable human data is present in the study, and de-identification was performed before analysis. Only researchers with authorization had access to the key that allowed data consultation.

This single-center study is part of a more extended research project, and the local Ethic Committee of Hospital Garcia de Orta reviewed and approved this study with the reference number: 05/2015 and 05/2016. The work was carried out in accordance with the Code of Ethics of the World Medical Association (Declaration of Helsinki). The authors declare that there are no conflicts of interest.

## 5. Conclusions

Previous data unequivocally showed a Th2 immune response at the local level in AA. The Th2 systemic immune response is characterized by elevated serum levels of Th2 cells, IL-5, IL-13, and IgE. In the present study, we were unable to detect systemic Th2 activation at the time of our sampling. 

## Figures and Tables

**Table 1:** Patient’s demographics.

	NPA *n* = 17 (20%)	APA *n* = 39 (47%)	AGA *n* = 27 (33%)	*p* Value	Multiple Comparisons
Age (years)	34.41 ± 10.39	38.05 ± 15.95	39.33 ± 14.16	0.537 *	
Sex (F/M)	14/3	25/14	14/13	0.122 **	
Allergy (N/Y)	14/3	31/6	23/4	0.999 ***	
BMI	22.98 ± 3.13	25.54 ± 5.76	25.69 ± 3.34	0.192 *	
Clinics	48 (24–72)	24 (17,5–36)	36 (24–48)	0.007 ****^a^	APA ≠ AGA (*p* = 0.031) APA ≠ NPA (*p* = 0.024)

M—Male; F—female. N—No; Y—yes; BMI—Body Mass Index in Kg/m^2^. Clinics—time in hours until medical care. Results are presented as Mean ± SD or Median (Q1–Q3). Sex in absolute numbers. * One-Way ANOVA. ** Qui-Square test. *** Qui-Square test by Monte Carlo simulation. **** Kruskal–Wallis test. a—Statistically significant differences at a 5% significance level.

**Table 2:** Hematologic parameters and appendicular histology.

	NPA	APA	AGA	*p* Value	Multiple Comparisons
WBC	12.25 ± 2.87	14.15 ± 4.44	14.40 ± 3.78	*p* = 0.134 **	
Neutrophils	8.23 (7.49–8.12)	11.59 (6.87–14.83)	11.75 (9.57–14.16)	*p* = 0.047 *^a^	AGA ≠ NPA (*p* = 0.046)
Lymphocytes	2.49 ± 0.98	1.98 ± 0.78	1.48 ± 0.63	*p* = 0.000 **^a^	AGA ≠ APA (*p* = 0.031); AGA ≠ NPA (*p* = 0.000)
Eosinophils	0.13 (0.04–0.22)	0.08 (0.03–0.15)	0.03 (0.02–0.07)	*p* = 0.003 *^a^	AGA ≠ APA (*p* = 0.025); AGA ≠ APA (*p* = 0.004)
Basophils	0.04 (0.03–0.05)	0.05 (0.03–0.08)	0.03 (0.02–0.04)	*p* = 0.03 *^a^	AGA ≠ APA (*p* = 0.002)
CRP	2.36 (0.54–7.00)	1.13 (0.70–4.20)	9.70 (4.00–13.50)	*p* = 0.001 *^a^	AGA ≠ APA (*p* = 0.001)

WBC—White Blood Count—Expressed in absolute numbers × 10^9^/L. Neutrophils—Expressed in absolute numbers × 10^9^/L. Lymphocytes—Expressed in absolute numbers × 10^9^/L. Eosinophils—Expressed in absolute numbers × 10^9^/L. Basophils—Expressed in absolute numbers × 10^9^/L. CRP—C-Reactive-protein expressed in mg/dL. Results are presented as Mean ± SD or Median (Q1–Q3). * Kruskal–Wallis test. ** One-Way ANOVA. ^a^ Statistically significant differences at a 5% significance level.

**Table 3:** Maturation of CD4 T cells in peripheral blood (PB) and appendicular histology.

	NPA	APA	AGA	*p* Value	Multiple Comparisons
Naïve TCD4	29.84 ± 12.26	26.39 ± 8.95	30.53 ± 11.33	*p* = 0.0689 **	
CM TCD4	22.78 ± 6.16	34.19 ± 9.16	19.52 ± 5.60	*p* = 0.003 **^a^	AGA ≠ APA *p* = 0.004); APA ≠ NPA (*p* = 0.035)
EM TCD4	38.72 ± 8.47	34.51 ± 8.27	35.50 ± 11.67	*p* = 0.711 **	
EMRA	8.68 ± 6.81	4.90 ± 4.72	14.07 ± 7.55	*p* = 0.025 **^a^	AGA ≠ APA (*p* = 0.020)

Results are presented as Mean ± SD. ** One-Way ANOVA. ^a^ Statistically significant differences at a 5% significance level.

**Table 4:** Helper T cells in PB and appendicular histology.

	NPA	APA	AGA	*p* Value	Multiple Comparisons
Th1	25.64 ± 7.97	27.85 ± 10.88	13.91 ± 9.56	*p* = 0.037 **^a^	AGA ≠ APA (*p* = 0.033)
Th2	69.86 ± 7.91	67.62 ± 12.99	84.42 ± 10.75	*p* = 0.028 **^a^	AGA ≠ APA (*p* = 0.025)
Th17	1.51 (0.69–5.12)	1.77 (013–5.04)	1.06 (0.45–2.38)	*p* = 0.580 *	

Results are presented in % Mean ± SD or Median (Q1–Q3). * Kruskal–Wallis test. ** One-Way ANOVA. ^a^ Statistically significant differences at a 5% significance level.

**Table 5:** Cytokines and IgE blood levels.

	NPA	APA	AGA	*p* Value	Multiple Comparisons
IL-4	6.82 (0.73–9.05)	6.25 (2.89–8.84)	10.34 (7.50–14.55)	*p* = 0.033 *^a^	AGA ≠ NPA (*p* = 0.032); AGA ≠ APA (*p* = 0.018)
IL-5	14.38 (12–20.52)	11.79 (9.40–19.07)	13.05 (10.76–18.79)	*p* = 0.282 *	
IL-13	0.13 (0.04–0.28)	0.14 (0.08–0.26)	0.11 (0.03–0.22)	*p* = 0.805 *	
IgE	35 (16–46)	23 (10–91)	47.5 (14.00–85.00)	*p* = 0.768 *	

Cytokines results are presented in pg/mL. IgE results are presented as IU/mL. Results are presented in Median (Q1–Q3). * Kruskal–Wallis test. ^a^ Statistically significant differences at a 5% significance level.

**Table 6:** Relationship between Th2 blood response parameters.

	IL-4	IL-5	IL-13	IgE
Th2	0.336	−0.089	−0.213	
IL-4		0.813 **	0.135	0.111
IL-5			−0.085	0.043
IL-13				0.293

Spearman correlation coefficient results. ** Correlation is significant at the 0.01 level (2-tailed).

## Data Availability

The original contributions presented in this study are included in the article/Supplementary Material. Further inquiries can be directed to the corresponding author.

## Supplementary Materials

The following supporting information can be downloaded at https://www.mdpi.com/article/10.3390/ijms27020733/s1.
