# Supplementary material for: Characterization of Th2 Serum Immune Response in Acute Appendicitis
Source: Int J Mol Sci. 2026 Jan 11;27(2):733. doi: 10.3390/ijms27020733 (PMC12841471; doi:10.3390/ijms27020733)
Supplement: Supplementary file 1 [file ijms-27-00733-s001.zip › Suplementary File S2 Gating Strategy.pdf]

Supplementary Material S2 – Representative images of the gating strategies used to identify immune cell populations obtained using multiparametric flow cytometry analysis of peripheral blood samples. A) T and B cells. Based on morphologic parameters, lymphocytes were discriminated (SSC-A versus FSC-A) and doublets were excluded from the analysis (FSC-H versus FSC-A). Gated on lymphocytes, T cells were identified by the expression of CD3 molecules and absence of CD19 expression. On the other hand, B cells were identified as CD3- and CD19+. **B) NK cells.** Like (A), NK cells were identified, within lymphocytes, by the expression of CD56 molecule and absence of CD3, CD19 and CD20 molecules. **C) CD4 T cells maturation.** Lymphocytes were discriminated as (A). Gated on single-cell lymphocytes; T cells were selected by the expression of CD3 molecule. Subsequently, CD4 T and CD8 T cells, as well as double positive (DP, CD4+CD8+) and double negative (DN, CD4-CD8-) T cells were classified based on the expression of CD4 and CD8 molecules. To assess the maturation state of CD4 T cells, the presence or absence of the CD45RA and CCR7 molecules were used to identify naïve cells (CD45RA+ CCR7+), central memory cells (CD45- CCR7+), effector memory cells (CD45RA- CCR7-) and effector cells (CD45RA+ CCR7-). **D) Regulatory T cells.** Initial selection of T cells was equal to (C). Gated on T cells, CD4 T cells were identified combining the double expression of CD3 and CD4 molecules. Within CD4 T cells, Treg cells were identified through the expression of CCR4 and CD25 molecules, and low or absent expression of CD127 molecule. Gated on Treg cells, it was also analyzed the expression of CD45RO molecule to assess the memory state, memory (CD45RO+) and naïve (CD45RO-) Treg cells.

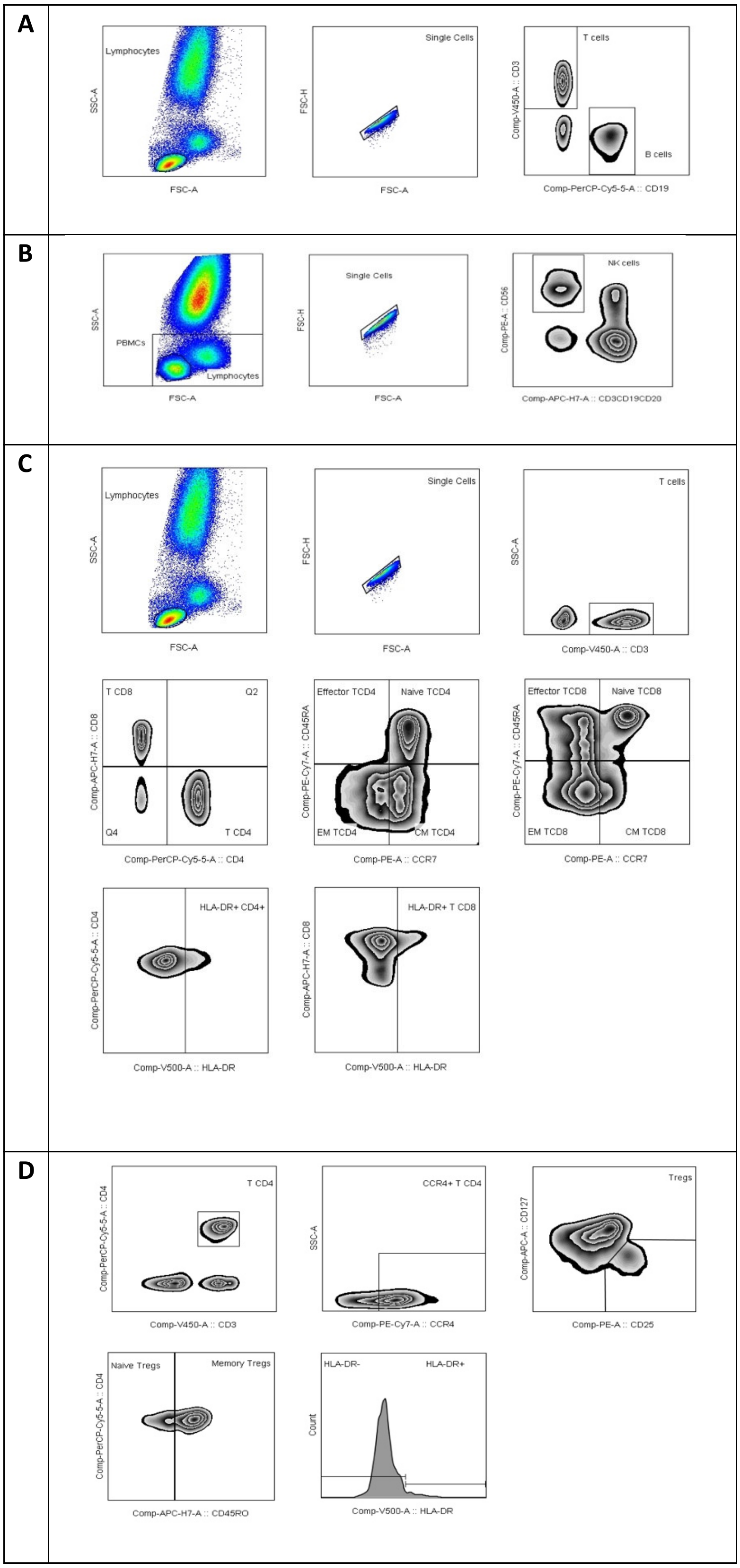
A
Lymphocytes
SSC-A
FSC-A
Single Cells
FSC-H
FSC-A
T cells
Comp-V450-A :: CD3
B cells
Comp-PerCP-Cy5-5-A :: CD19
B
SSC-A
PBMCs
Lymphocytes
FSC-A
Single Cells
FSC-H
FSC-A
NK cells
Comp-PE-A :: CD56
Comp-APC-H7-A :: CD3CD19CD20
C
Lymphocytes
SSC-A
FSC-A
Single Cells
FSC-H
FSC-A
T cells
SSC-A
Comp-V450-A :: CD3
T CD8
Q2
Q4
T CD4
Comp-APC-H7-A :: CD8
Comp-PerCP-Cy5-5-A :: CD4
Effector TCD4
Naive TCD4
EM TCD4
CM TCD4
Comp-PE-Cy7-A :: CD45RA
Comp-PE-A :: CCR7
Effector TCD8
Naive TCD8
EM TCD8
CM TCD8
Comp-PE-Cy7-A :: CD45RA
Comp-PE-A :: CCR7
HLA-DR+ CD4+
Comp-PerCP-Cy5-5-A :: CD4
Comp-V500-A :: HLA-DR
HLA-DR+ T CD8
Comp-APC-H7-A :: CD8
Comp-V500-A :: HLA-DR
D
T CD4
Comp-PerCP-Cy5-5-A :: CD4
Comp-V450-A :: CD3
CCR4+ T CD4
SSC-A
Comp-PE-Cy7-A :: CCR4
Tregs
Comp-APC-A :: CD127
Comp-PE-A :: CD25
Naive Tregs
Memory Tregs
Comp-PerCP-Cy5-5-A :: CD4
Comp-APC-H7-A :: CD45RO
HLA-DR-
HLA-DR+
Count
Comp-V500-A :: HLA-DR
