# Supplementary material for: Characterization of Th2 Serum Immune Response in Acute Appendicitis
Source: Int J Mol Sci. 2026 Jan 11;27(2):733. doi: 10.3390/ijms27020733 (PMC12841471; doi:10.3390/ijms27020733)
Supplement: Supplementary file 1 [file ijms-27-00733-s001.zip › Suplementary Table S1. Lymphocytes cells in PB.pdf]

Supplemental Table S1. Lymphocytes cells in PB and Appendicular Histology

	NPA	APA	AGA	*p* value
T cells	76.24±4.9	68.35±11.44	73.37±4.48	p=0.242 **
B cells	8.43±5.69	13.33±6.29	11.73±6.97	p=0.390 **
NK cells	8.02(7.31-13.90)	12.20(4.59-17.40)	7.79(3.66-11.50)	p=0.375 *

p.

PB- Peripheral Blood

NPA- Non-Pathological Appendice; APA- Acute Phlegmonous Appendicitis;

AGA-Acute Gangrenous Appendicitis;

Results are presented in % (Mean±SD or Median (Q1-Q3)

*** Kruskal-Wallis test. **One Way-ANOVA.**

$p<0.05$ is considered significant
