# Supplementary material for: Characterization of Th2 Serum Immune Response in Acute Appendicitis
Source: Int J Mol Sci. 2026 Jan 11;27(2):733. doi: 10.3390/ijms27020733 (PMC12841471; doi:10.3390/ijms27020733)
Supplement: Supplementary file 1 [file ijms-27-00733-s001.zip › Suplementary Table S2. T cells in PB.pdf]

Supplemental Table S2. T cells in PB and Appendicular Histology

	NPA	APA	AGA	*p* value
TCD4+	68.10±5.30	59.63±10.76	56.38±12.94	p=0.193 **
TCD4 + TCD8+	0.41±0.29	1.05±0.93	1.34±0.83	p=0.176 **
TCD8 +	26.54±5.41	30.24±8.06	30.03±13.85	p=0.444 **
TCD4 - TCD8-	4.92±2.24	0.08±5.81	8.27±3.54	p=0.277 **
TCD4 HLA- DR+	68(67.20-70.70)	62.30(54.20-67.10)	59.35(47.60-66.00)	p=0.854 *
TCD8 HLA- DR+	26.40(23.20-26.40)	28.80(23.40-37.60)	29.80(24.00-42.80)	p=0.519 *

PB- Peripheral Blood

NPA- Non-Pathological Appendice; APA- Acute Phlegmonous Appendicitis;

AGA-Acute Gangrenous Appendicitis;

Results are presented in % (Mean±SD or Median(Q1-Q3))

*** Kruskal-Wallis test. **One Way-ANOVA.**

$p<0.05$ is considered significant
