# Supplementary material for: Characterization of Th2 Serum Immune Response in Acute Appendicitis
Source: Int J Mol Sci. 2026 Jan 11;27(2):733. doi: 10.3390/ijms27020733 (PMC12841471; doi:10.3390/ijms27020733)
Supplement: Supplementary file 1 [file ijms-27-00733-s001.zip › Suplementary Table S3. Regulatory T cells.pdf]

Supplemental Table S3. Regulatory T cells in PB and Appendicular Histology

	NPA	APA	AGA	*p* value
Treg	2.66±1.09	2.39±1.33	1.95±1.56	p=0.684 **
Naïve	0.47±0.26	0.34±0.24	0.41±0.36	p=0.702 **
Memory	2.16(1.50-2.48)	2.04(0.92-2.99)	0.93(0.64-3.10)	p=0.550 *
HLA-DR+	0.26±0.15	0.34±0.28	0.14±0.008	p=0.141 **

PB- Peripheral Blood

NPA- Non-Pathological Appendice; APA- Acute Phlegmonous Appendicitis;

AGA-Acute Gangrenous Appendicitis;

Results are presented in % (Mean±SD or Median(Q1-Q3))

*** Kruskal-Wallis test. **One Way-ANOVA.**

$p<0.05$ is considered significant
