# Supplementary material for: Characterization of Th2 Serum Immune Response in Acute Appendicitis
Source: Int J Mol Sci. 2026 Jan 11;27(2):733. doi: 10.3390/ijms27020733 (PMC12841471; doi:10.3390/ijms27020733)
Supplement: Supplementary file 1 [file ijms-27-00733-s001.zip › Supplementary File S1 Ab.pdf]

Supplementary Material S1 – Monoclonal antibodies used for multiparametric flow cytometry analysis.

Ab	Conjugate	Clone	Brand	Cat#
CD3	V450	ICHT1	BD Horizon	561416
CD3	APC-H7	SK7	BD Biosciences	560176
CD4	PerCp-Cy5.5	OKT4	Biolegend	317428
CD8	APC-H7	HIT8a	BD Biosciences	641400
CD19	PerCp-Cy5.5	HIB19	Biolegend	302230
CD19	APC-H7	SJ2501	BD Biosciences	560177
CD20	APC-H7	2H7	BD Pharmingen	560734
CD25	PE	M-A251	BD Pharmingen	555432
CD45RA	PE/Cy7	5H9	BD Biosciences	561216
CD45RO	APC-H7	UCHL1	BD Biosciences	561137
CD56	PE	B159	Biolegend	318306
CD127	AF647	HIL-7R-M21	BD Biosciences	558598
CXCR3	PE	IC6/CXCR3	BD Biosciences	550633
CCR4	PE/Cy7	IG1	BD Biosciences	561034
CCR6	PE/Cy7	11A9	BD Biosciences	560620
CCR7	PE	150503	BD Biosciences	560765
HLA-DR	V500	G46-6	BD Biosciences	561224
